# Factors related to the suppression of the antitumour immune response in female dogs with inflammatory mammary carcinoma

**DOI:** 10.1371/journal.pone.0267648

**Published:** 2022-05-05

**Authors:** Karine Araújo Damasceno, Aline Michelle dos Santos-Conceição, Laís Pereira Silva, Thiago Marconi de Souza Cardoso, Carlos Humberto da Costa Vieira-Filho, Samantha Hellen Santos Figuerêdo, Emanoel Martins-Filho, Barbra Gabriela Oliveira de Faria, João Moreira da Costa-Neto, Geovanni Dantas Cassali, Alessandra Estrela-Lima

**Affiliations:** 1 Laboratory of Experimental Pathology, Gonçalo Moniz Institute, Salvador, Bahia, Brazil; 2 Research Center on Mammary Oncology NPqOM/HOSPMEV, Federal University of Bahia, Salvador, Bahia, Brazil; 3 Postgraduate Program in Animal Science in the Tropics, Federal University of Bahia, Salvador, Bahia, Brazil; 4 Laboratory of Clinical Research, Gonçalo Moniz Institute, Salvador, Bahia, Brazil; 5 Laboratory of Comparative Pathology, Department of General Pathology, Federal University of Minas Gerais, Belo Horizonte, Minas Gerais, Brazil; University of Bologna, ITALY

## Abstract

Inflammatory mammary carcinoma (IMC), a neoplasia affecting women and female dogs, is considered an aggressive cancer with high metastatic potential and a low survival rate. Studies focused on the tumour microenvironment indicate that the aggressive behaviour of this tumour is primarily correlated with immunological factors as well as inflammation. The objective of this study was to analyse the possible strategies used by the tumour cells to suppress the immune response in female dogs with IMC. Forty-six female dogs were divided into three groups: control (C, n = 10), IMC (n = 14) and mammary carcinoma (MC, n = 22). Clinical-pathological evaluations, survival at follow-up, immunophenotyping of leukocytes in peripheral blood and tumours, and immunohistochemical evaluation of CD4^+^, granzyme B, perforin and FAS-L were performed. Clinical and pathological results showed a higher frequency of the primary form of neoplasia, solid arrays of tumor cells and a lower survival rate in the IMC group (30 days). Morphometric analysis of inflammatory infiltrate revealed more lymphocytes and macrophages in the IMC group. Immunophenotyping analysis of peripheral blood revealed a higher frequency of CD8^+^ T-cells (p = 0.0017), a lower frequency of CD4^+^ T-cells (p <0.0001), and significantly higher mean MHCI and MHCII CD14^+^ fluorescence intensity in the IMC group (p = 0.038 and p = 0.0117, respectively). The immunohistochemical evaluation of tumour sections showed fewer FAS-L-positive inflammatory cells in the IMC group. These results suggest the important contribution of CD8^+^ T-cells, macrophages and FAS-L in the aggressiveness of IMC.

## Introduction

Inflammatory mammary carcinoma (IMC) represents a great challenge in veterinary medicine due to this cancer’s aggressive characteristics, including rapid growth, high metastatic potential, non-responsiveness to treatment and low survival rate [1, 2]. Due to its aggressivity, IMC has been the target of many investigations attempting to determine cell lineage, the role of Cox-2 in the behaviour of tumour cells, treatment response, survival, the presence of hormonal receptors and histopathological and immunological characterization [3–9].

Inflammatory breast cancer in women, like IMC in female dogs, is the most aggressive and lethal manifestation of primary epithelial mammary cancer [10–14]. This neoplasm is uncommon and accounts for approximately 7.6% of all female dogs with mammary tumours [8]. However, increasing frequencies of IMC have been observed by some researchers over the last decade [15, 16]. Affected dogs have an average survival of only 60 days after clinical diagnosis and, to date, existing treatments are palliative in nature, while surgical excision is contraindicated [5, 15].

In neoplastic development, the innate immune system recognizes neoplastic cells as abnormal due to some factors produced by them that promote signals to the immune system, stimulating it to destroy them [17]. After activation, cytotoxic lymphocytes induce apoptosis of tumor cells by releasing perforin (a protein homologous to complement protein C9, present in cytoplasmic granules of NK and CD8 T cells, induces the formation of transmembrane pores in target cells, allowing entry of granzymes), granzymes (serine protease released by exocytosis and in contact with the target cell cleaves and activates caspases that induce apoptosis of these cells) and FAS/FAS-L (FAS ligand—induces cell death when FAS binds to FAS-L expressed on activated CD8 cells) [18–20].

It is believed that, in certain situations, neoplastic cells can develop mechanisms of resistance to the immune response [16]. However, the immune escape pathways and the cross-regulation of these pathways in the course of aggressive canine tumour progression, such as in inflammatory carcinoma, remains unclear [21, 22].

Improving the understanding of the mechanisms involved in immune evasion, as well as in the antitumour immune response, are fundamental for characterizing IMC and developing new therapeutic approaches. The objective of this study was to analyze the tumour microenvironment and the possible strategies used by tumour cells to suppress the immune response in female dogs with inflammatory mammary carcinoma.

## Material and methods

### Study sample

Forty-six female dogs, purebred or mixed-breed, aged from 8–18 years, were selected between October 2015 and October 2016 following admission to the Veterinary Hospital of the Federal University of Bahia (HOSPMEV-UFBA).

Based on clinical evaluations, female dogs were divided into three experimental groups: the inflammatory mammary carcinoma (IMC) group (n = 14) composed by female dogs with specific clinical presentation of inflammatory carcinoma and not submitted to previous treatment; the mammary carcinoma (MC) (n = 22) group composed by female dogs with any histological subtype of carcinoma presenting metastases in regional lymph nodes or distant metastases, representing clinical stage IV and V, respectively, were defined based on TNM system previously described by Owen, 1980 [23]; the control group (n = 10) consisted of clinically healthy female dogs with no history of mammary cancer or no comorbidity. The use of anti-inflammatory medication 30 days before the mastectomy was also considered an exclusion criterion. The histological classification was defined according to Consensus for the Diagnosis, Prognosis and Treatment of Canine Mammary Tumors [24].

All experimental procedures were performed in accordance with the guidelines established by the National Council for the Control of Animal Experimentation (CONCEA). This study was approved by the Institutional Review Board for Animal Experimentation at the Federal University of Bahia (CEUA-UFBA no. 13/16).

### Clinical staging

All female dogs were submitted to a comprehensive clinical examination. Clinical anamnesis was accomplished, involving a detailed evaluation of physiological parameters, clinical history and reproductive records, along with biochemical and hematological analysis, and thoracic radiography and abdominal ultrasound to identify metastasis. In the MC and IMC groups, macroscopic evaluations included the characterization of tumor features (size, signs of inflammation and/or ulceration) and location. as well as inspection of regional lymph nodes by palpation. In swollen lymph nodes, fine-needle aspiration cytology was performed to detect metastasis and to establish differential diagnosis.

Clinical staging criteria were adapted from the WHO TNM classification system [23] for canine mammary tumors. Stage is determined from information on the size of tumor (T), involvement of regional lymph node (N) and the presence or absence of distant metastases (M). All female dogs belonging to the IMC group was stage V, regardless of the diagnosis of regional or distant metastasis. The MC group consisted of female dogs with tumors of any size with lymph node metastasis (stage IV) or distant metastases (stage V).

After obtaining a clinical suspicion of MC, female dogs underwent unilateral radical mastectomy with the removal of regional lymph nodes. Immediately after surgery (in the MC group) or after death (in the IMC group), the mammary chain and regional lymph nodes were sent to the Laboratory of Veterinary Pathology at the Federal University of Bahia (LPV-UFBA) for macroscopic and microscopic, characterization of the tumor and confirmation of metastasis, respectively.

### Tumour samples, histological classification and histological grading

Five representative fragments of the intratumour and peripheral tumour areas were randomly selected and removed from each tumour. Necrotic areas were excluded. Tumour fragments measuring 1.0×1.0 cm, taken from the affected mammary gland, were submitted to histological, morphological/morphometric and immunophenotypic analysis.

All histopathological samples were fixed in 10% buffered formalin, embedded in paraffin and cut into 4 μm sections. Histopathological diagnosis was based on haematoxylin and eosin (H&E) staining in accordance with the WHO standardization of the Consensus for the Diagnosis, Prognosis and Treatment of Canine Mammary Tumors [24].

Histological grade was defined according to the Nottingham histological grading method [25] by evaluating the percentage of tubular formation, nuclear pleomorphism, and mitotic count using 4 μm HE stained tissue sections. For nuclear pleomorphism, the score #1 was used when the nuclei were small, with little increase in size in comparison with normal mammary epithelial cells, and had regular outlines and a uniformity of nuclear chromatin. The score of #2 was assigned when the cells were larger than normal, had open vesicular nuclei with visible nucleoli, and showed moderate variability in size and shape. The score #3 corresponded to marked variation in size and shape, especially when very large and bizarre nuclei were vesicular with prominent and often multiple nucleoli. For evaluation of mitotic activity, 10 neoplasm fields were evaluated, without necrosis or artifacts, using a BX40 Olympus microscope, 40× objective, 10× ocular field number, 22 mm field view of diameter, and 0.55 mm in the sample level fields. Each high-power field (HPF) corresponds to an area of 0.237 mm^2^. All typical and atypical mitoses were counted in 10 fields (i.e., 2.37 mm^2^ of area). Tumor up to 7 mitotic figures was scored as 1 point, 8–16 mitotic figures as 2 points and more than 17 mitotic figures as 3 points. Final histological grade was obtained by adding up the scores #1, #2 and #3 and classified as follows: i) grade I: 3–5 points, well differentiated; ii) grade II: 6–7 points, moderately differentiated and grade III: 8–9 points, poorly differentiated.

### Morphological/Morphometric analysis of tumour inflammatory infiltrate

Morphological alterations in the total inflammatory infiltrate were classified according distribution and intensity [26]. Morphometric analysis was carried out in eight selected "hot spots" representative of both peripheral and intratumour areas, and the number of inflammatory cells were assessed. Images were captured using an oil immersion 100× objective (a digital camera was coupled to an Olympus BX-40 microscope) and SPOT version 3.4.5 software. Inflammatory cells were characterized by image analysis (Corel Draw software version 7.468) and identified based on morphological features. The total number of inflammatory cells was obtained by quantification of the eight analysed fields. The cut-off was determined based on the mean cell number, and two intervals were considered in accordance with lymphocytic infiltrate intensity: i) discrete or moderate (< 600 lymphocytes), or ii) intense (≥ 600 lymphocytes).

### Immunophenotyping of canine whole blood leukocytes by flow cytometry

To determine the frequencies of whole cells on peripheral blood, 36 samples, collected in EDTA (2 mL) were lysed using ACK lyse buffer (BD Biosciences CA) during 10 minutes and washed two times with PBS 1X (Sigma, St. Louis, MO) at 300 x g. The cells were adjusted at a concentration of 5 x 10^5^ cells/mL in polypropylene tubes to stain with monoclonal antibodies hosted on sheep with reactivity against canine antigens, α-CD3 (clone CA17.2A12), α-CD4 (clone YKIX 302.9), α-CD8 (clone YCATE55.9), MHC-I (clone 2G5) and MHC-II (clone YKIX334.2) in order to determine lymphocytes and antigen presentation molecules over monocytes and B cells it was used. To determine monocytes were used monoclonal antibodies hosted on sheep with reactivity against canine antigens α-CD14 (clone TÜK4), α-CD21 antibody (clone CA2.1D6) to determine B-lymphocytes and α-CD45 antibody (clone CA12.10C12) to determine the common leukocytes antigen following previous protocol instruction [27]. All monoclonal antibodies used from Bio-Rad Laboratories (California, USA). Flow cytometric measurements were performed on a FACS Calibur instrument (Becton Dickinson, San Jose, CA, USA). Analysis of flow cytometry was performed using the Cell Quest^™^ Software package. A minimum of 50,000 gated events from each sample were collected in a FACS Fortessa flow cytometer (Becton Dickinson and Company, Franklin Lakes, NJ) and analysed using the FlowJo 7.6.5 program.

### Immunophenotyping of tumour lymphocytic infiltrate by flow cytometry

Tumour fragments (n = 36) representative of peri/intra-tumour areas were submitted to a modified method of immunophenotypic analysis by flow cytometry [28]: tissue fragments were immersed in cold RPMI 1640 medium (5 mL) in a petri plate and placed on ice for maceration. The tumours fragments were macerated using the BD^™^ Medimachine system using medicons of 50μm at 10 minutes (automated, mechanical disaggregation of solid animal standardization) followed by filtration on Falcon of 50μm to cells purification. Cells from tumours were washed two times with PBS 1X and quantified to 1 X 10^6^ cells/mL harvested in PBS till cell stained process.

To stain tumour cells were used monoclonal antibodies hosted in sheep with canine reactivity α-CD4-RPE 1:320 (rat-IgG2a, clone YKIX302.9) and α-CD8-AlexaFluor 647 1:40 (rat-IgG1, clone YCATE55.9) (Bio-Rad Laboratories California, USA). The combinations and dilutions of mAbs were employed according to Araújo et al. [27]. It was used for negative controls, mAbs of the same isotype (IgG1a), produced in the same species and obtained from the same manufacturer, were used.

Flow cytometric measurements were performed on a FACS Calibur instrument (Becton Dickinson, San Jose, CA, USA). Data analysis was performed by first gating lymphocyte populations based on forward scatter (FSC) versus side scatter (SSC) properties. Immunophenotypic features were analysed on dual colour FL1/FITC versus FL2/R-PE dot plots. Analysis of flow cytometry was performed using the Cell Quest^™^ Software package. A minimum of 100,000 gated events from each sample were collected in a FACS Fortessa flow cytometer (Becton Dickinson and Company, Franklin Lakes, NJ) and analysed using the FlowJo 7.6.5 program. The results were expressed as the frequency of positive cells among gated lymphocytes, or as the percentage of CD4^+^, CD8+ T-cells or the CD4+/CD8+ T-cell ratio among gated T-cells.

### Immunohistochemistry

To perform immunohistochemical studies, the following mAbs were used to detect: CD4, granzyme B, perforin and FAS-L (Table 1). Sections (4 μm) were cut from one representative block of each tumour sample. Tissue sections were deparaffinized in xylene and examined using the NovoLink Max Polymer Detection System (Leica Biosystems^®^).

**Table 1:** Antibody sources, manufacturers, clones, dilutions, incubation times, and antigen retrieval methods.

Antibody	Antibody source	Clone	Diluition	Incubation time	Antigenal retrieval
CD4	Sino Biological^®^	M22	1:50	30 minutes	HIER, citrate pH 6
FAS-L	Arigo^®^	Policlonal	1:50	16 hours	HIER, citrate pH 6
Granzyme B	Arigo^®^	Policlonal	Ready to use	1 hour	HIER, citrate pH 6
Perforin	Arigo^®^	DG9	1:100	1 hour	Enzymatic Pepsin

HIER = heat-induced epitope retrieval. Previously tested mammary gland samples were used as positive controls; negative controls were obtained by replacement of the primary antibody by IgG. Detection system used was NovoLink detection system (Leica Biosystems, Buffalo Grove, IL).

CD4, granzyme B and FAS-L were subjected to heat-induced antigen retrieval with sodium citrate buffer (pH 6.0) in a water bath at 98°C for 22 min. Perforin was subjected to antigen retrieval and digestion at 37°C for 30 min with 2% pepsin in 10 μM HCl (pH 2.0). Endogenous peroxidase activity was blocked with 3% hydrogen peroxidase in methanol. Slides were incubated for 30 min with the anti-CD4 monoclonal antibody, for 1 hour with the antibodies against granzyme B and perforin, and then overnight at 4°C with the anti-FAS-L monoclonal antibody. It was used a polymeric system for antibody detection (Novolink^™^ Max Polymer Detection System). Finally, diaminobenzidine (DAB) was used as a chromogen, and sections were counterstained with Mayer’s haematoxylin.

Negative controls were prepared by replacing the primary antibody with normal serum. Lymph nodes were used as positive controls for CD4, granzyme B and perforin, and testicular tissues were used as positive controls for FAS-L. To evaluate the effectiveness of cytotoxic cells, percentages of cells positively labelled with granzyme B and perforin were calculated.

The immunohistochemical analysis was carried out following the same procedures described above in the morphometric analysis section. The percentages of lymphocytes positive for CD4 and CD8 were calculated, and the percentages of mononuclear cells positive for FASL, granzyme B and perforin were determined from the total mononuclear inflammatory cell population. All histological assessments were performed under a conventional light microscope (Olympus–BX41) with a 20×, 40× or 60× magnification.

### Follow-up and survival

The animals with MC were evaluated monthly, while dogs with IMC were submitted to weekly clinical examinations involving laboratory testing (erythrogram, leukogram and serum biochemistry for urea, creatinine, ALT and ALP), which were repeated until death. Throughout the survival period, all animals were monitored to evaluate the progression or regression of inflammatory processes associated with neoplasia and each animal’s general condition. Overall survival rate, expressed in days, was defined as the period between primary tumour detection and death in the IMC group, and as the time between surgical excision of the primary tumor and the date of death or end of follow-up. Dogs’ owners were instructed to contact the veterinary staff responsible for the project in case of animals have any severe pain or suffering. Veterinarians were prepared to perform prompt evaluation, treatment and define whether the animal should undergo a more specific treatment or even humanitarian euthanasia (with the owner’s consent).

Dogs that died during the follow-up period were necropsied at the Veterinary Pathology Laboratory to determine the cause of death and to assess for possible metastasis. Animals were considered censored when they did not return for periodic clinical evaluation and contact was lost or when they died from other causes unrelated to the tumor.

### Statistical analysis

Statistical analysis was performed using the GraphPad Prism 6.0 software package (San Diego, CA, USA). Comparative analyses of nonparametric data between groups (C, IMC and MC) were performed by the Kruskal–Wallis test followed by Dunn’s post-test to compare all sample pairs. For parametric data, a one-way analysis of variance (ANOVA), followed by the Student’s t-test were used. Pearson’s correlation was used to evaluate associations between the survival (days) in the IMC group and specific cell phenotypic features (CD4^+^, CD8^+^, CD4^+^/CD8^+^ T-cell ratio and MHC-I expression by monocytes), as well as the frequency of macrophages in inflammatory infiltrate. Survival curves were estimated using the Kaplan–Meier estimation method, followed by log-rank testing. In all cases, the differences were considered significant when p< 0.05.

## Results

### Clinical and pathological features

**Fig 1:**
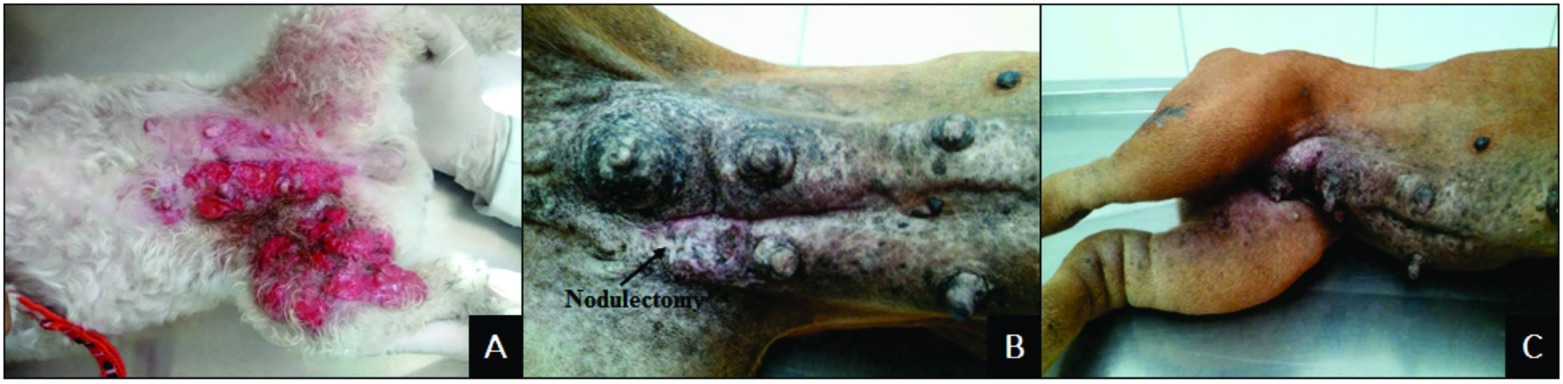
Macroscopic features of inflammatory mammary carcinoma in female dogs. A) Plaque-like growth in the left mammary chain extending to the inner face of the pelvic limb presenting diffuse erythema and containing extensive erosion and ulceration areas. B) Mammary chains with plaque growth after nodulectomy. C) Intense edema of the limbs.

Dogs in the IMC group had a mean age of 10.7 years, whiles those in the MC group presented a mean age of 10.8 years. Clinical evaluations determined seven cases diagnosed as primary IMC (7/14), four as secondary (4/14) and three as secondary post-surgical IMC (3/14) (Fig 1B).

The tumour plaques present in the IMC group measured between 10–20 centimeters in length. In seven animals, the neoplastic plaque presented areas of ulceration (7/14) (Fig 1A), while the nodules in the other seven animals were intact (7/14). In addition, three of these animals presented edema of the limbs (Fig 1C). In the MC group, thirteen animals (13/22) presented nodules larger than 5 cm, while nine had nodules smaller than 5 cm. Twelve of these animals (12/22) presented ulcerated nodules, while the other ten had intact nodules (10/22). The histopathological analysis of the IMC group identified the following carcinoma subtypes: solid (7/14), papillary (3/14), anaplastic (2/14), micropapillary (1/14) and pleomorphic lobular (1/14).

**Fig 2:**
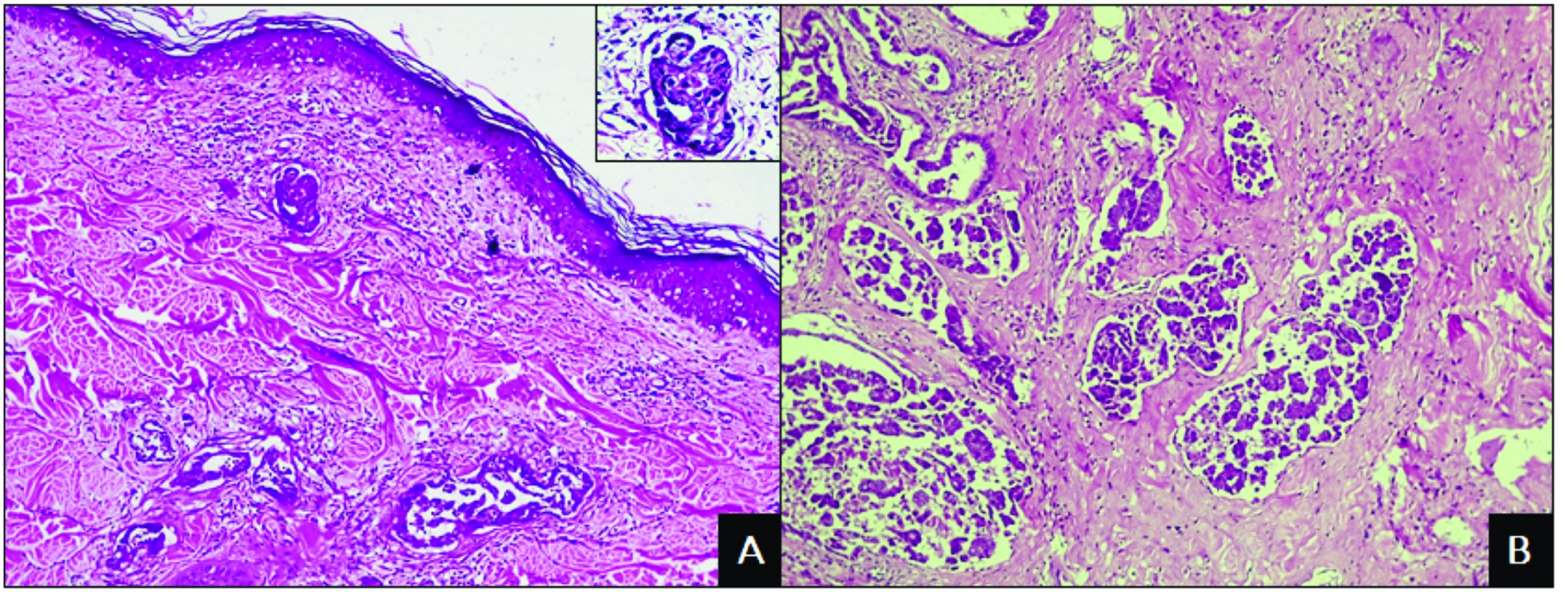
Photomicrographs of inflammatory mammary carcinoma in female dogs. A) Proliferation of neoplastic cells in the superficial dermis associated with peritumoral inflammatory infiltrate. HE. Obj. 10x. Detail: Highly pleomorphic cells. HE. Obj. 40x. B) Proliferation of neoplastic cells invading the mammary parenchyma with developed stromal support. HE. Obj. 10x.

The presence of marked cellular and nuclear pleomorphism, anisocytosis, anisokaryosis, eosinophilic cytoplasm and a mitotic index of III was observed in most IMC cases (Fig 2A). Additionally, the inflammatory infiltrate was characterized as mononuclear, of moderate to severe intensity, with a predominance of lymphocytes and macrophages. Some tumours revealed areas of comedocarcinoma and/or cribriform arrangement, in addition to squamous metaplasia. All cases presented lymph vessel invasion, primarily in the superficial dermis, dermis and peritumoural areas (Fig 2B). Metastases were diagnosed in live animals by imaging exams, aspiration cytology and histopathological analysis of regional lymph nodes after mastectomy and after death by necroscopic examination. The main sites were lymph nodes (14/14) and lungs (7/14), but metastases were also detected in the intercostal muscles, spleen, liver, adrenal glands, pancreas, skin, pericardium, diaphragm and trachea.

In the histopathological examination of the MC group, fifteen animals (15/22) presented mixed tumour carcinoma, five (5/22) had solid carcinoma and two (2/22) had tubular carcinoma. Lymph node metastasis was present in all cases, and pulmonary metastases occurred in seven animals, with results indicating advanced tumour stages (Stage IV and V).

### Morphologic and morphometric analysis

In the IMC group, a comparative analysis of inflammatory infiltrate morphology showed a multifocal distribution in eight animals (8/14), diffuse distribution in six animals (6/14), moderate intensity in five animals (5/14) and severe intensity in the other nine (9/14). In the MC group, multifocal distribution was observed in 15 animals (15/22) and diffuse distribution in seven (7/22), with discrete intensity seen in eight animals (8/22), moderate in eight (8/22) and severe intensity in the remaining six (6/22).

The morphometric findings were not significantly different among the groups; however, the female dogs in the IMC group presented more lymphocytes and macrophages, as well as fewer plasma cells, neutrophils and eosinophils, than the MC group (Table 2).

**Table 2:** Morphometric analysis of the inflammatory tumour infiltrates of female dogs with inflammatory mammary carcinoma or mammary carcinoma (advanced stage).

Cells	IMC	MC	p*
Lymphocytes	770.7± 279.2	593.2 ± 372.7	0.13
Macrophages	249.8 ± 172.6	215.5 ± 154.1	0.53
Plasmocytes	91.3 ± 46.6	150.6 ± 138.7	0.13
Neutrophils	82.2 ± 132.4	159.4 ± 222.0	0.24
Eosinophils	7.4 ± 8.7	19.68 ± 24.20	0.07
Total	1198.28 ± 388.40	1138 ± 399.3	0.60

* Unpaired t test

### Immunophenotypic analysis of peripheral blood leukocytes

**Fig 3:**
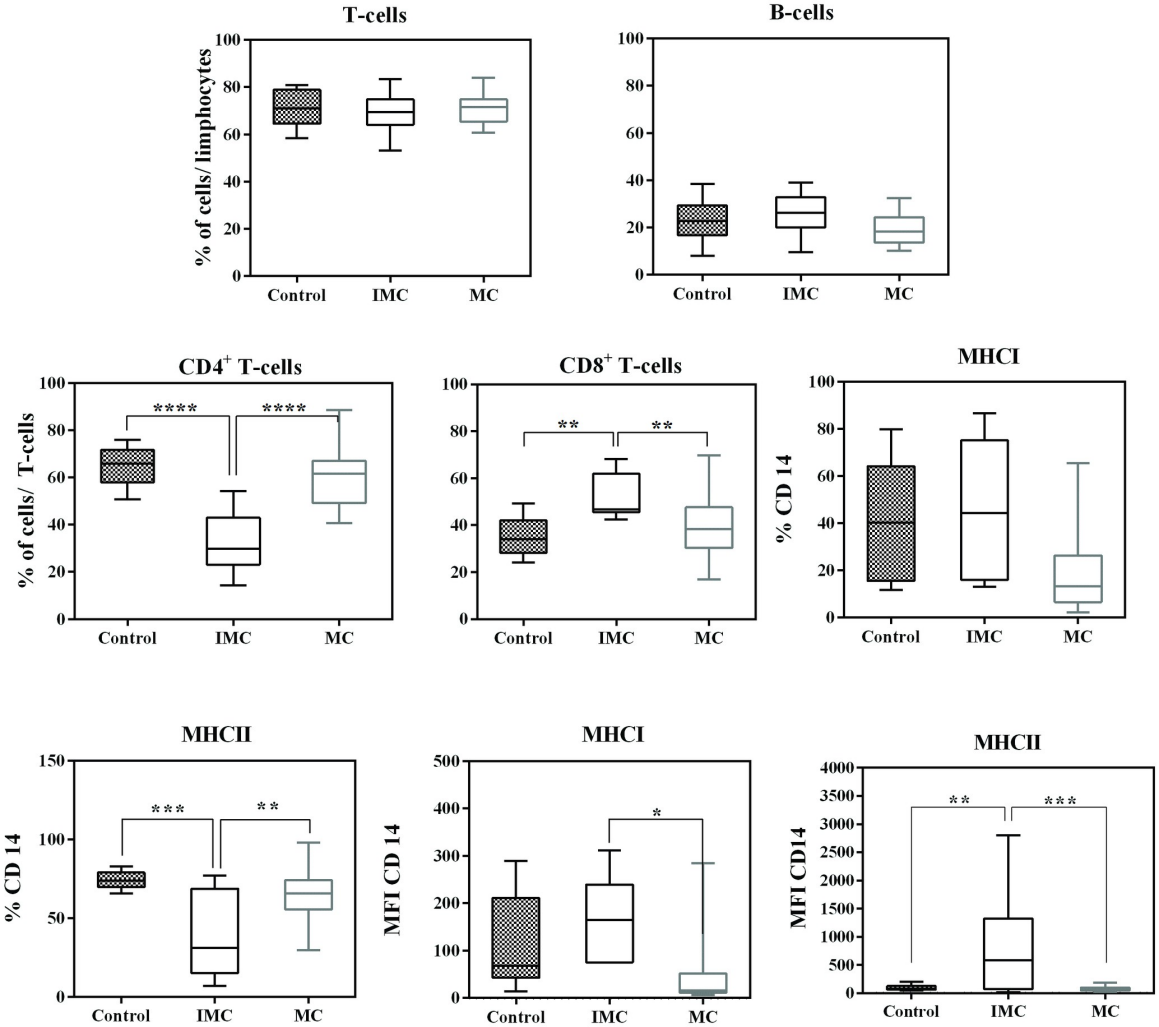
Leucocyte subsets in the peripheral blood of female dogs with mammary carcinoma. Results are shown in box plot format, depicting frequencies of CD4+, CD8+, CD21+ T-lymphocytes, as well as CD14+CD45+, CD14+ MHC-I+ CD14+ MHC-II+ monocytes, and MFI MHCI and MFI MHC-II. Significant differences (P<0.05) are indicated by *, P<0.01 is indicated by **, P< 0.001 is indicated by *** and P< 0.0001 is indicated by ****.

The frequencies of T lymphocytes were 71.4 ± 7.8% for the C group, 68.8 ± 8.6% for the IMC group and 71± 6.6% for the MC group. The B lymphocyte frequency was 23.0 ± 8.7% in the C group, 25.8 ± 9% in the IMC group and 19.3 ± 6.7% in the MC group. No significant differences were seen in these lymphocyte populations between these three groups (Fig 3).

The subpopulation analysis of T-cells in group C indicated 64.7 ± 8.2% for CD4^+^ T-cells and 35.3 ± 8.2% for CD8^+^ T-cells. In the IMC group, the percentages were 32.7 ± 13.5% for CD4^+^ T-cells and 52 ± 8.8% for CD8^+^ T-cells. In the MC group, the percentages were 60.3 ± 12.8% for CD4^+^ T-cells and 39.77 ± 12.7 for CD8^+^ T-cells. Here, the IMC group presented significant difference compared to groups C and MC (Fig 3). The percentage of CD4^+^ T-cells in the IMC group was significantly lower (p <0.0001), while the CD8^+^ T-cell frequency was higher (p = 0.0033) than in the other two groups investigated.

The percentage of CD14^+^ MHC-I^+^ cells observed in the control group was 40.6 ± 25.9%, with a mean fluorescence intensity (MFI) of 121.8 ± 98.0, and the frequency of CD14^+^ MHC-II^+^ cells was 74.2 ± 5.8%, with an MFI of 100.7 ± 52.6. In the IMC group, the percentage of CD14^+^ MHC-I^+^ cells was 45.6.6 ± 28.2%, with an MFI of 159 ± 69.5, and the frequency of CD14^+^ MHC-II^+^ cells was 38.6 ± 27%, with an MFI of 772.1 ± 867. In the MC group, the percentage of CD14^+^ MHC-I^+^ cells was 20.1 ± 20.2%, with an MFI of 48.7 ± 73.2, and the frequency of CD14^+^ MHC-II^+^ cells was 51.9 ± 29.5, with an MFI of 77.94 ± 43. The frequency of CD14^+^ MHC-I^+^ cell markers was significantly higher in the IMC group (p = 0.010), while CD14^+^ MHCII^+^ was significantly lower (p <0.0001). However, the MFI of the CD14^+^ MHCII^+^ cells was higher in the IMC group (p = 0.0003) than MC, and that in the MC was significantly lower than in the C group (p<0.05). Finally, the MFI of the CD14^+^ MHCI^+^ cells was significantly higher (p = 0.0003) in the IMC group compared to MC (Fig 3).

### Flow cytometric immunophenotyping of tumour infiltrating lymphocytes

**Fig 4:**
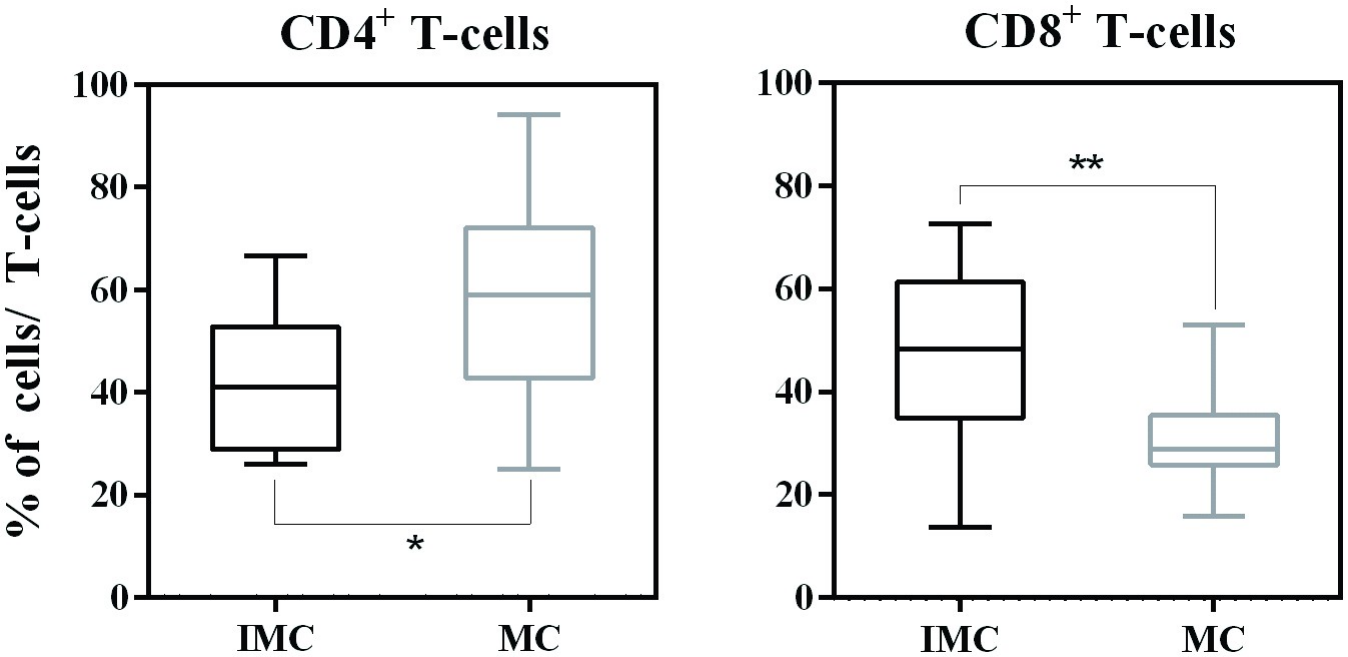
Immunophenotypic profile of tumour-infiltrating lymphocytes in female dogs with mammary carcinoma. Analysis of tumour-infiltrating T-cell subsets from inflammatory mammary carcinoma (IMC) and mammary carcinoma (MC) samples. Data are expressed as the percentage of positive cells among either CD4+ or CD8+ T-cells.

In the IMC group, the frequency of CD4^+^ T-cells was 41.8 ± 13.7%, and the frequency of CD8^+^ T-cells was 46.5± 18%. In the MC group, the percentage of CD4^+^ T-cells was 58.2± 19%, and the percentage of CD8^+^ T-cells was 31 ± 9.5%. The CD4^+^ and CD8^+^ T-cell frequency results were consistent with results in peripheral blood, which revealed a significantly lower percentage of CD4^+^ T-cells and a higher frequency of CD8^+^ T-cells in the IMC group (Fig 4).

### Immunohistochemistry

**Fig 5:**
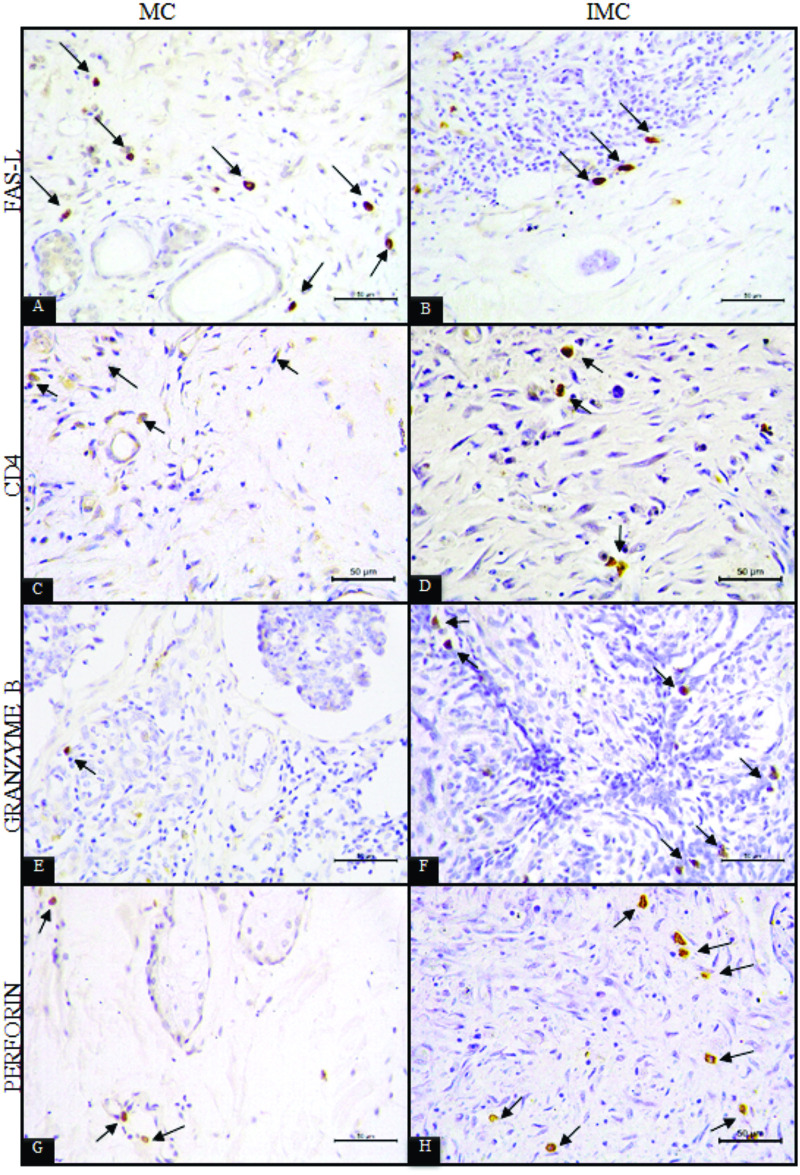
Photomicrography of immunohistochemically-stained sections. A) inflammatory cells positive for cytoplasmic expression of FAS-L in peritumoral inflammatory infiltrates of mammary carcinoma (MC) tissue. B) Inflammatory cells positive for cytoplasmic expression of FAS-L in peritumoral inflammatory infiltrates of inflammatory mammary carcinoma (IMC) tissue. C) Inflammatory cells positive for membrane expression of CD4 in the peritumoral inflammatory infiltrate of mammary carcinoma (MC) tissue. D) Inflammatory cells positive for membrane expression of CD4 in the peritumoral inflammatory infiltrate of inflammatory mammary carcinoma (IMC) tissue. E) Inflammatory cells positive for cytoplasmic expression of granzyme B in the peritumoral inflammatory infiltrate of mammary carcinoma (MC) tissue. F) Inflammatory cells positive for cytoplasmic expression of granzyme B in the peritumoral inflammatory infiltrate of inflammatory mammary carcinoma (IMC) tissue. G) Inflammatory cells positive for cytoplasmic expression of perforin in the peritumoral inflammatory infiltrate of mammary carcinoma (MC) tissue. H) Inflammatory cells positive for cytoplasmic expression of granzyme B in the peritumoral inflammatory infiltrate of inflammatory mammary carcinoma (IMC) tissue.

In the peritumoural inflammatory infiltrate of the IMC group, 15.8 ± 4.6% of cells presented cytoplasmic expression of FAS-L (Fig 5B), 5.7 ± 2.4% expressed granzyme B (Fig 5F), 6.1 ± 2.5% expressed perforin (Fig 5H) and membrane expression of CD4 was identified in 10.4 ± 5.6% (Fig 5D).

In the MC group, 30.7 ± 10.8% of the cells presented cytoplasmic expression of FAS-L (Fig 5A), 2.2 ± 1.3% expressed granzyme B (Fig 5E), 7.6 ± 1.8% expressed perforin (Fig 5G), and 20.9 ± 4.7% of the inflammatory cells had membrane expression of CD4 (Fig 5C).

**Fig 6:**
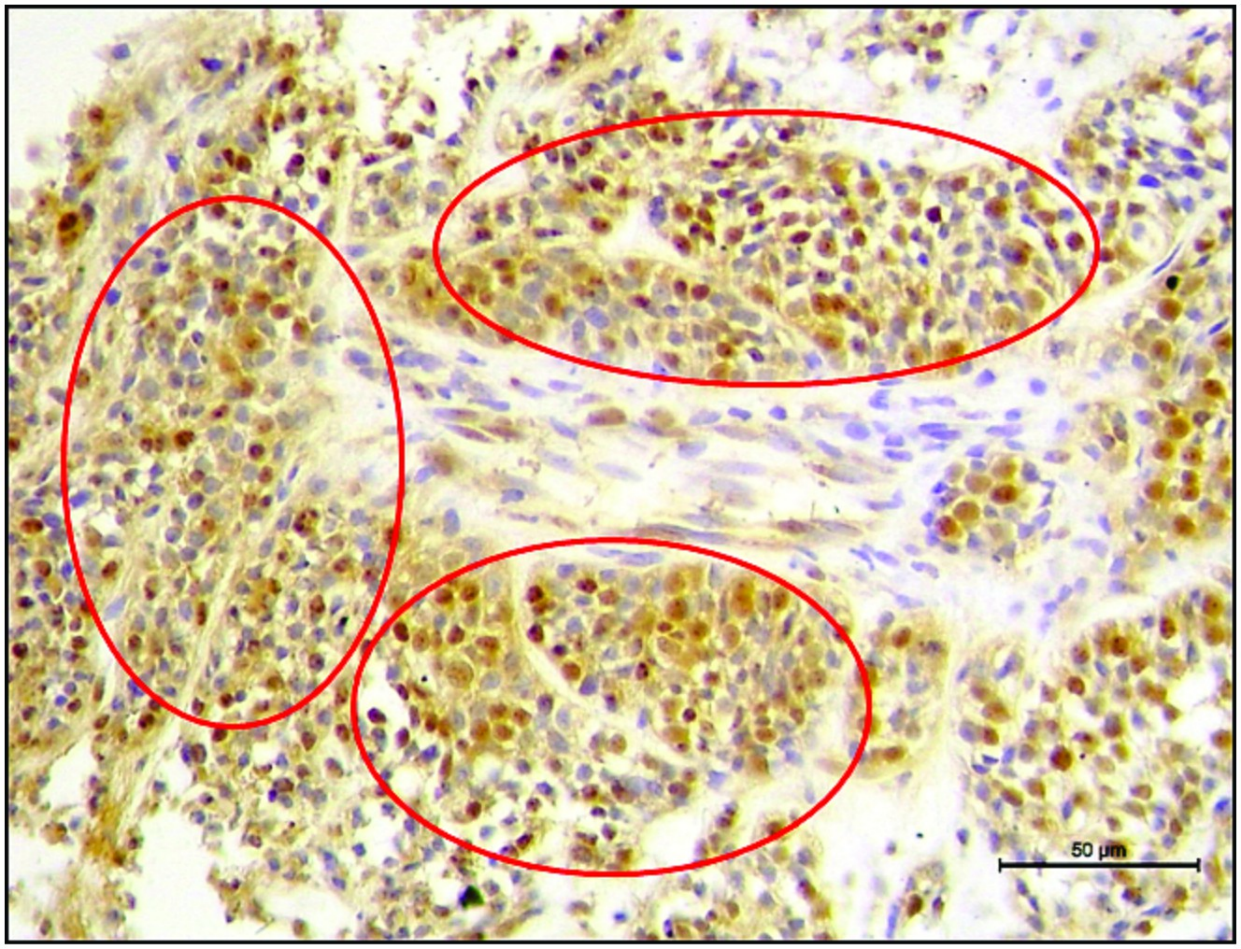
Photomicrography of immunohistochemical analysis. Circles indicate foci of tumor cells with positive cytoplasmic and nuclear expression of FAS-L in the mammary carcinomas of female dogs.

The expression of FAS-L by inflammatory cells was higher in the MC group than in IMC (p = 0.0003). In some animals in the MC group, membrane and nuclear FAS-L expression were observed in malignant epithelial cells (Fig 6). Granzyme B expression was significantly higher in the IMC group (p < .0001), and CD4 expression was statistically lower (p<0.0001) in relation to the MC group. No significant statistical differences were observed in perforin expression.

### Survival curve comparison

**Fig 7:**
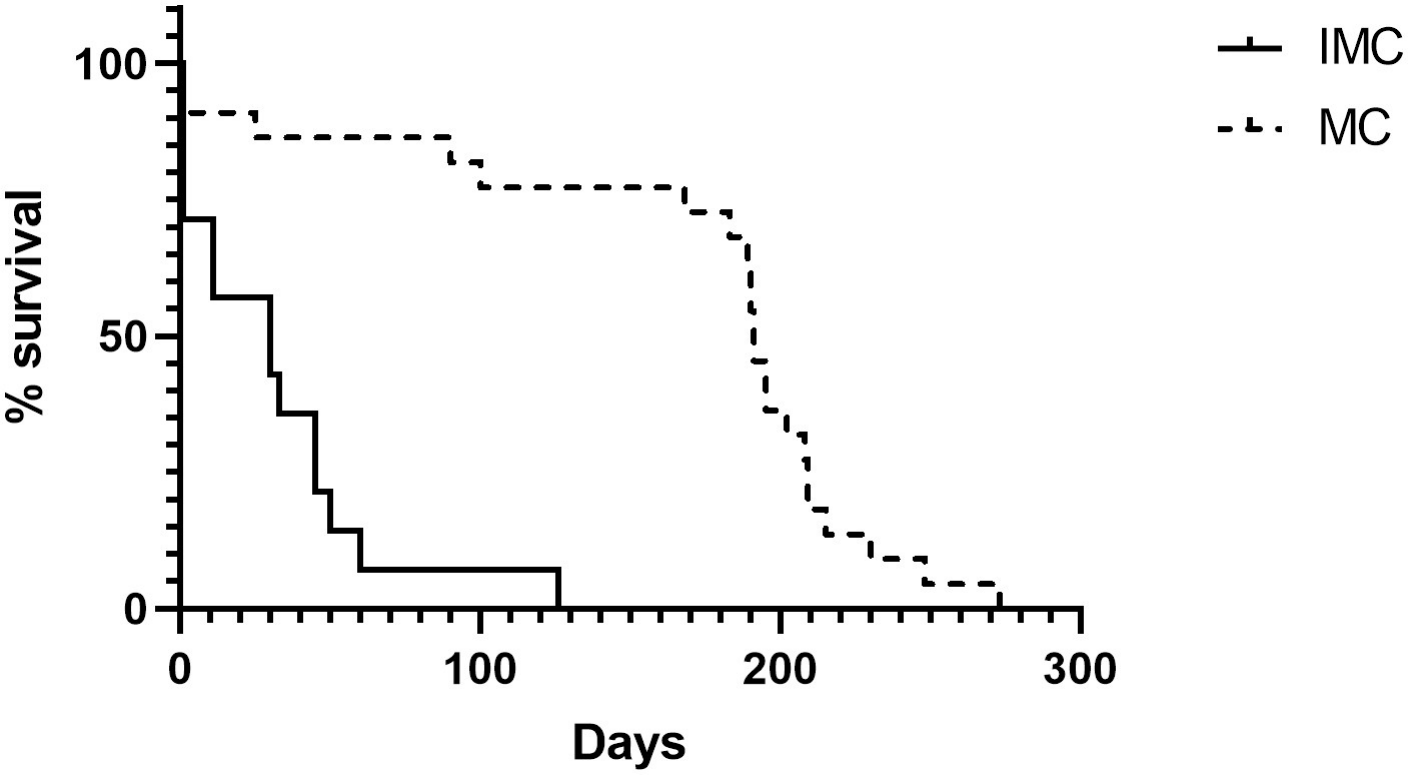
Survival rates of dogs with mammary carcinoma. Kaplan-Meier survival curves for animals in IMC and MC groups. Significant differences at p = 0.001 are denoted by ***.

The maximum overall survival period observed for the IMC group was 126 days, versus 273 days in the MC group. The median survival in the IMC group was 30 days compared to 191 days in the MC group. Six animals in total (02 from the MC group and 04 from the IMC group) were submitted to humane euthanasia at the time of diagnosis (with the owner’s consent), given the evident condition of suffering due to the advanced stage of the disease. No animal was considered censored as it was possible to monitor all animals during the study period. Thus, a significantly lower survival rate was found in female dogs affected by IMC (p<0.0001; Fig 7).

The correlation between survival rate and the number of infiltrating macrophages in the tumours from female dogs with IMC was significantly negative (r = -0.52 and p = 0.03). No statistically significant correlations were identified between survival and the number of lymphocytes, plasma cells, neutrophils or eosinophils.

## Discussion

In this study, the mean age of the female dogs evaluated was 10 years, which is consistent with reports in the literature [8, 29, 30]. More animals were affected by the primary subtype, which is classically associated with the more aggressive form of this cancer, as well as with a worse prognosis [8]. Interestingly, animals with secondary post-surgical IMC presented more severe symptoms than those with primary IMC, which resulted in the euthanasia of these animals at the time of diagnosis. This finding could be plausible if the owners did not take their animals for evaluation at the onset of symptoms and sought treatment only after the disease progressed and became irreversible. In this study, the mean survival of the IMC group (30 days) was shorter than the mean survival of 60 days reported in the literature [5, 15].

Anaplastic carcinoma, although described as the most frequent subtype in this study, was only observed in two cases, with solid carcinoma predominating [6, 31, 32]. Reports in the literature, in consonance with the present study, indicate the presence of a predominantly mononuclear inflammatory infiltrate, with the highest frequency of cells being lymphocytes, followed by macrophages. It has been suggested by some authors that the tumour lymphocytic infiltrate is related to increased invasiveness and metastatic potential, as well as decreased survival rates, which are factors that determine a worse prognosis, as was observed in the IMC cases [22, 33, 34].

Due to the greater number of macrophages in the IMC group than the MC group, and the inverse correlation of the frequency of this cell type with survival rate, the results herein support the hypothesis that macrophages participate in the aggressiveness of inflammatory carcinoma in female dogs. Infiltrating macrophages in the tumour play a dual role in the neoplastic microenvironment based on the availability of oxygen and tumour progression. M1 (pro-inflammatory) cells are recruited by inflammatory mediators in early stages of neoplastic growth although favorable outcomes are currently questioned [35–37]. Conversely, in advanced tumours, classic macrophages are converted to alternative macrophages (M2 pro-tumour) by suppressor cells or cytokines These cells release cytokines and chemokines that promote immunoregulation, tissue remodelling and angiogenesis, which inhibits the immune response and promotes metastasis, thus creating more aggressive and metastatic tumours [38–41]. Peripheral blood leukocyte immunophenotyping data from the CD14^+^ cell frequency evaluation showed a higher percentage of monocytes expressing surface MHC-I molecules in the IMC group. MHC molecules are crucial for lymphocyte activation and the adaptive immune response [42], so these findings are consistent with the percentages of CD8^+^ and CD4^+^ cells observed in this study. Low MHC-I expression was also found in metastatic non-inflammatory carcinomas [43], which strengthens the hypothesis that MC cells employ mechanisms to decrease MHC-I expression to evade the immune response [43–45].

Regarding CD14 cells, our results suggest that IMC tumour cells are more antigenic than MC tumour cells and thus recruit a greater number of inflammatory cells to the tumour site. This affects the modulation of the tumour microenvironment and a consequent increase in the invasive potential of neoplastic cells, although the underlying mechanism is poorly understood [43].

In the immunophenotypic evaluation of peripheral blood, low frequencies of CD4^+^ T-cells and high frequencies of CD8^+^ T-cells were demonstrated in the IMC group relative to the MC and C groups. When the infiltrating lymphocytes in the tumour were evaluated by immunophenotyping, variations similar to those found in peripheral blood were observed, suggesting the recruitment of these cells to the tumour site.

Another study [46] performed peripheral blood immunophenotyping in female dogs with non-inflammatory carcinomas and reported findings similar to those of the present study in animals with non-metastatic tumours, which have a good prognosis. Contrary results were found in the present study, which showed that female dogs had worse prognosis. Other studies have reported altered CD4^+^ and CD8^+^ T-cell frequencies in blood and tumours of dogs with mammary neoplasms; however, these findings remain controversial [47–50].

For a more effective antitumour immune response, the activation of CD8^+^ T-cells is necessary, occurring via the presentation of antigens or the action of cytokines released by CD4^+^ T-cells [18]. Due to the production of these immunosuppressive factors, cytotoxic T-cells may become inefficient, or other subtypes of CD8^+^ T-cells with regulatory characteristics may be selected. These processes may help explain the greater aggressiveness, invasiveness and worse prognosis of inflammatory carcinomas [42, 51, 52]. A higher percentage of CD8^+^ T-cells does not represent greater efficiency in the antitumour response, since the neoplastic cells in the tumour microenvironment can inhibit the effector action of lymphocytes [53].

The observed granzyme-B data may translate into a lower efficiency of CD8^+^ T-cells, since perforin release is required for the formation of transmembrane pores in target cells to guide the granzyme in its cytotoxic action [54, 55].

The positive labelling of FAS-L allowed for the observation of different behaviours between inflammatory and advanced stage mammary carcinomas. A higher number of positive inflammatory cells was observed in the MC group, in some cases with many positively labelled neoplastic cells.

Conversely, a smaller number of FAS-L-positive inflammatory cells was observed in the IMC group, and no FAS-L labelling was observed in neoplastic cells. FAS-L functions in the immunological hemostasis against autoimmunity via apoptosis of immune cells, particularly CD8^+^ T-cells [56]. Since FAS-L is poorly expressed in cytotoxic cells, suppressor cells, such as Tregs, myeloid precursor cells and macrophages, are recruited to attain immune balance, resulting in the accumulation of inflammatory cells at the tumour site, which promotes the escape of tumour cells from the immune response [20, 56, 57]. The low expression of FAS-L in inflammatory cells in IMC indicates dysregulation in the immune response, as well as increased inflammatory infiltrate and more active suppression mechanisms.

Macrophages, CD8^+^ T-cells and other mononuclear cells expressing FAS-L were associated with tumour aggressiveness and lower survival rates, which may indicate the participation of these cells in promoting a suppressor immune state in the tumour microenvironment in dogs with inflammatory mammary carcinoma. The positive labelling of FAS-L illustrated distinct differences in behaviour between IMC and advanced stage MC. The MC animals presented greater numbers of FAS-L^+^ cells and some neoplastic cells were also found to be FAS-L^+^, which provides evidence of a possible mechanism to evade the immune response, caused by an increase in apoptosis of cytotoxic cells, as demonstrated by the lower frequency of CD8^+^ T-cells in the tumours in this group. The accumulation of inflammatory cells promotes the release of excess pro-inflammatory factors that increase the chemoattraction of myeloid-derived cells and macrophages. These cells promote tumour invasion and metastasis through the production of matrix metalloproteinases and various chemoattractants, and also express TGF-β and IL-10, which stimulates the activity of regulatory T-cells and tumour-associated macrophages (TAMs) [58, 59].

## Conclusions

IMC and MC are aggressive types of canine and human tumors and herein we showed that IMC canine tumours present, in terms of peripheral blood and tissue infiltrates, a higher CD8 activity, mediated by granzyme B and perforin and that could lead to more tissue cellular matrix destruction and consequently metastasis. The crosstalk of CD8, CD4, and tissular macrophages in that IMC group, with higher MHC-I and lower FasL, promotes the inflammatory microenvironment maintenance and a lower survival compared to MC group. All together those data showed that the inflammatory profile observed on IMC correlated to the worst outcome, even compared to MC, showing more aggressive characteristics. Besides, there is a lack of knowledge of how the inflammatory and anti-inflammatory profiles could control the growth or dissemination of tumors cells on IMC.

## Supporting information

S1 Table Clinicopathological, immunohistochemical, morphometric data of tumor-associated inflammation and immunophenotyping (blood and tumor) of animals in the control group (C, n = 10), inflammatory mammary carcinoma (BMI, n = 14) and mammary carcinoma (MC, n = 22). (XLSX)
